# Correlation between High PD-L1 and EMT/Invasive Genes Expression and Reduced Recurrence-Free Survival in Blood-Circulating Tumor Cells from Patients with Non-Muscle-Invasive Bladder Cancer

**DOI:** 10.3390/cancers13235989

**Published:** 2021-11-28

**Authors:** Maria Beatrice Morelli, Consuelo Amantini, Jacopo Adolfo Rossi de Vermandois, Marilena Gubbiotti, Antonella Giannantoni, Ettore Mearini, Federica Maggi, Massimo Nabissi, Oliviero Marinelli, Matteo Santoni, Alessia Cimadamore, Rodolfo Montironi, Giorgio Santoni

**Affiliations:** 1 School of Pharmacy, Section of Experimental Medicine, University of Camerino, 62032 Camerino, Italy; federica.maggi@uniroma1.it (F.M.); massimo.nabissi@unicam.it (M.N.); oliviero.marinelli@unicam.it (O.M.); 2 School of Biosciences and Veterinary Medicine, University of Camerino, 62032 Camerino, Italy; consuelo.amantini@unicam.it; 3 Urologic and Andrologic Clinics, University of Perugia, 05100 Perugia, Italy; ja.rossidevermandois@aospterni.it (J.A.R.d.V.); ettore.mearini@unipg.it (E.M.); 4 Department of Urology, San Donato Hospital, 52100 Arezzo, Italy; marilena.gubbiotti@uslsudest.toscana.it; 5 Department of Medical and Surgical Sciences, University of Siena, 53100 Siena, Italy; antonella.giannantoni@unisi.it; 6 Neurosciences, Functional and Surgical Urology Unit, Santa Maria alle Scotte Hospital, 53100 Siena, Italy; 7 Department of Molecular Medicine, University Sapienza, 00185 Rome, Italy; 8 Oncology Unit, Macerata Hospital, 62100 Macerata, Italy; mattymo@alice.it; 9 Section of Pathological Anatomy, Department of Biomedical Sciences and Public Health School of Medicine, Polytechnic University of Marche Region, Umberto I Hospitals, 60121 Ancona, Italy; a.cimadamore@staff.univpm.it; 10 Molecular Medicine and Cell Therapy Foundation, Department of Clinical and Molecular Sciences, Polytechnic University of Marche Region, 60126 Ancona, Italy; r.montironi@univpm.it

**Keywords:** NMIBC, CTC, immunotherapy, PD-L1, EMT, TWIST1, TIMP2, RFS

## Abstract

**Simple Summary:**

The correlation between immune checkpoint-programmed death-ligand 1 (PD-L1) marker and epithelial–mesenchymal transition (EMT) status may help to identify potential biomarkers for the use of immune checkpoint blockades and other immunotherapy approaches in non-muscle invasive bladder cancer (NMIBC) recurrent patients. The aim of our study was to assess to potential use of *PD-L1*, *TWIST1*, *ZEB1*, *VIMENTIN* and *TIMP2* mRNA expression as prognostic biomarkers.

**Abstract:**

Background: PD-L1 represents a crucial immune checkpoint molecule in the tumor microenvironment, identified as a key target for cancer immunotherapy. A correlation between *PD-L1* and EMT-related genes expression in various human cancers has been suggested. Methods: By ScreenCell filtration, digital droplet PCR and confocal microscopy analysis, we aimed to investigate the expression of *PD-L1* and EMT/invasive genes (*TWIST1*, *ZEB1*, *VIMENTIN*, *TIMP2*) in circulating tumor cells (CTCs) collected from the blood of non-muscle-invasive bladder cancer (NMIBC) patients, assessing the prognostic value of these biomarkers in the disease. Welchs’ test and Mann–Whitney U test, correlation index, Kaplan–Meier, Univariate and Multivariate Cox hazard proportional analysis were used. Results: Higher *PD-L1*, *TIMP2* and *VIM* mRNA levels were found in pT1 compared to pTa NMIBC. As evaluated by Kaplan–Meier and Univariate and Multivariate Cox analysis, enhancement of *PD-L1*, *TWIST1* and *TIMP2* expression reduces the recurrent free survival in NMIBC patients. Conclusions: High *PD-L1*, *TWIST1* and *TIMP2* mRNAs mark the recurrent-NMIBC patients and by reducing the RFS represent negative prognostic biomarkers in these patients.

## 1. Introduction

Over 400,000 patients are newly diagnosed with bladder cancer (BC) every year [1]. Three quarters of them have tumors that do not invade the detrusor muscle, which are described as non-muscle-invasive bladder cancers (NMIBCs) [2]. NMIBCs confined to the mucosa or invading the lamina propria are classified as stage Ta or T1, respectively, accordingly to the TNM classification system, whereas flat high-grade tumors confined to the mucosa are classified as CIS (Tis) [2]. NMIBCs are characterized by a relatively good prognosis, allowing most NMIBC patients to undergo only local treatment to prevent tumor recurrence and progression to muscle-invasive BC (MIBCs) that presents worse prognosis for metastatic disease. Treatment of NMIBC patients is based on the risk of tumor recurrence and/or progression [2]. Patients with high risk NMIBC are treated with transurethral resection of bladder tumor (TURBT) followed in some cases by Bacille Calmette Guerin (BCG) instillations. Standard treatment for patients with tumors that recur also after adequate BCG treatment is the surgical removal of the bladder (cystectomy), which is associated to high risk of complications, mortality and reduction of patient’s quality life [3,4].

The immune checkpoint marker, programmed death-1 (PD-1), and its ligand, programmed death ligand-1 (PD-L1, CD274, B7-H1), have recently attracted interest in the field of cancer immunology [5]. PD-L1 is frequently overexpressed in tumors [5]. By binding to PD-1, it may be possible to inhibit the activation of T lymphocytes in order to evade the host immune response, preventing tumors from cytotoxic T lymphocyte-induced killing. PD-L1 also interacts with B7.1 to further suppress the tumor antigen-induced activation of cytotoxic T lymphocytes. The blockade of this pathway using specific inhibitors such as pembrolizumab, nivolumab or atezolizumab could enhance the cytotoxicity of T cells in the tumor environment and substantially increase long-term survival in different cancers [5,6].

BCs have been characterized as a tumor group in which the immunological response is well conserved. *PD-L1* is expressed in BC and recent studies have suggested that it represents a mediator of stage progression [7], with BC patients expressing high *PD-L1* levels showing a poor prognosis and relapse-free survival. As of January 2020, the FDA approved pembrolizumab (anti-PD-1) for treating high risk BCG unresponsive NMIBC patients [8]. This phase II trial reported a 3-month clinical complete response in 44.6% of Tis patients, 41.7% of T1 and concomitant CIS patients, with 52.6% of complete responses lasting over 12 months and no progression to MIBC [8]. Moreover, in a comparable phase II trial (SWOG S1605) evaluating the PD-L1 inhibitor atezolizumab, a 3-month complete response rate in 41.1% of patients with Ta/T1 tumors and concomitant CIS (NCT02844816) was reported [9].

Recent evidence in cancers suggests a relationship between *PD-L1* and epithelial mesenchymal transition (EMT) gene expression [10,11]. EMT is a multi-step process involving the transition from an epithelial to mesenchymal phenotype in response to chemoresistance and is important for cancer metastasis and recurrence [12,13,14,15]. EMT upregulates *PD-L1* expression through the phosphoinositide 3-kinase/protein kinase B pathway in breast cancer [16]. Analyzing data from the Cancer Genome Atlas (TCGA) database, patients with positive *PD-L1* expression and EMT have a worse prognosis compared to those with positive *PD-L1* and negative EMT in head and neck squamous cell carcinoma [17]; moreover, a negative prognostic potential as well as high risk of recurrence and tumor progression have been reported in esophageal squamous cell carcinoma (ESCC) [11].

At present, biomarkers predictive of recurrence or progression for NMIBC are currently lacking. Liquid biopsy and, in particular, circulating tumor cells (CTCs) are currently under investigation to address this need [18,19]. CTCs are tumor cells that originate from a primary tumor, flowing through the bloodstream and circulating throughout the body, which may contribute to hematogenous metastasis [20]. Meta-analysis has shown that in MIBC patients CTCs are significantly associated with tumor progression and poor overall survival (OS) [21]. However, to date, blood CTCs have been understudied in NMIBC, although they are identified in these tumors.

Given the potential of CTCs for risk stratification and the lack of information relative to prognostic factors in NMIBC patients, we isolated CTCs in the blood from NMIBC patients undergoing TURBT, to evaluate the expression of *PD-L1* and its correlation with EMT markers by digital droplet PCR (ddPCR). We also investigated the correlation of these markers with clinic-pathological features and time to recurrence to determine their prognostic significance.

## 2. Materials and Methods

### 2.1. Patients and Ethic Statement

Peripheral blood was collected before TURBT from 50 patients with a diagnosis of NMIBC (Tis, Ta and T1) undergoing TURBT from 2018 to 2020 at the Urologic and Andrologic Clinics, University of Perugia, Perugia, Italy. The patients were heterogeneous with respect to their clinical course. All the patients or their guardians provided written informed consent according to the research proposals approved by the Ethical Committee CEAS UMBRIA (Ethic Committee approval code: URO009-3171/18). The use of patient data and CTCs for research purposes at the University of Perugia has been executed pursuant to Italian legislation and international standards.

### 2.2. CTC Blood Sample Processing

Peripheral blood was collected into ScreenCell blood collection tubes. 7.5 mL of each blood sample collected in a K2-EDTA was processed for 3 h using ScreenCell devices (Sarcelles, France) according to the manufacturer’s protocol. ScreenCell filters were washed with RPMI 1640 medium and Red Blood Lysis Buffer (MiltenyBiotec, Bologna, Italy). Then, enrichment filter-adherent CTCs were collected in RPMI medium and cell suspension filtered again. The ScreenCell microfiltration is an epitope-independent size-based device, able to capture all CTCs in the bloodstream. It has been used in CTC identification in rare tumors like hemangiopericytoma [22] as well as in more common tumors including bladder cancer [23].

### 2.3. RNA Extraction

Total RNA from isolated CTCs was extracted by using the Single Shot Cell Lysis Kit (BioRad, Hercules, CA, USA) according to the protocol.

### 2.4. Reverse Transcription

Total RNA was retrotranscribed by Iscript Advanced cDNA Synthesis kit (BioRad) and the resulting cDNA was used to preamplify each sample for all primers used in the gene expression analysis by SSOADvancedPreAmp Kit and PrimePCRPreAMP Assays (BioRad, Hercules, CA, USA).

### 2.5. Digital Droplet PCR (ddPCR)

The ddPCRSupermix for Probes (No dUTP) (BioRad) and the specific PrimePCR^TM^ddPCR^TM^ Expression Probe Assays conjugated with FAM or HEX fluorescent dyes (the same pool used in the preamplification step) (BioRad) were then used to perform the ddPCR. The following target genes were analyzed: *PD-L1*, *zinc finger E-box binding homebox 1 (ZEB1)*, *Twist family BHLH transcription factor 1 (TWIST1)*, *Vimentin (VIM) and the invasive gene TIMP Metallopeptidase Inhibitor 2 (TIMP2).* Data, normalized to *β-actin* concentration, were analyzed using the QuantaSoft Software (BioRad). Since some of the analyzed transcripts could also be expressed, although at low levels, in normal blood cells ddPCR analysis was carried out identifying the *CD3D*, *CD19*, *CD45*, *ICAM1*, *Beta2-microglobulin*, *ITGA2B/CD41*, *GYPA/CD235a* gene expression in NMIBC samples, comparing the results with those obtained from peripheral blood mononuclear cells from a normal human donor and taking them as negative threshold.

### 2.6. CTC Sample Staining

To further confirm the ddPCR data, CTCs were fixed in 4% paraformaldehyde for 5 min at room temperature. Then, cells were permeabilized by using 0.3% Triton X-100 in PBS for 15 min at room temperature. Then, the blocking solution (3% BSA, 0.3% Triton X-100 in PBS) was added for 60 min at room temperature. Staining was performed using mouse anti-human pan-Cytokeratin (C11) Ab (PanCK; 1:50, sc-8018, Santa Cruz Biotechnology, Heidelberg, Germany), anti-human CD45 Ab (1:50, #13917, Cell Signaling Technology, Danvers, MA, USA) and anti-human EpCAM (VU1D9) Ab (1:50, #2929, Cell Signaling Technology) followed by goat anti-mouse IgG H&L Ab (Alexa Fluor^®^ 594, 1:100, Abcam, Cambridge, UK), labeled with PureBlu™ DAPI (#1351303, BioRad) according to the manufacturer’s protocol. Slides were then analyzed under 60X magnification with C2 Plus confocal laser scanning microscope (Nikon Instruments, Firenze, Italy). Optimized emission detection bandwidths were configured by Zeiss Zen control software. Images were processed using NIS Element Imaging Software (Nikon Instruments, Firenze, Italy). Candidate CTCs were identified from top ranked cells by one of two trained technicians. In accordance with the manufacturer’s protocol, the definition for epithelial CTCs required cells to have a DAPI-positive nucleus with a diameter ≥4 μm, CK or EpCAM staining surrounding ≥50% of the nucleus, and the absence of staining in the counterstain channel (CD45).

### 2.7. Statistical Analysis

This was a pilot study. The analysis of frequency distribution was performed using a Chi-squared test selecting as expected frequencies: <60 for age; male category for sex; papillary for histology; pathological grade and stage. *p* < 0.05 was considered as statistically significant. Recurrence-free survival (RFS) was defined as the time from TURBT to the clinical or histological evidence of tumor recurrence. We determined, by Relative Operating Characteristic (ROC) curve analysis, the expression value for each analyzed gene that best discriminated between recurrence or not. In addition, the Welch’s *t*-test and Mann–Whitney U test were used to compare gene expression using GraphPad Prism version 7.00 for Windows, GraphPad Software (La Jolla, California, CA, USA, www.graphpad.com, accessed on 16 October 2021). *p* < 0.05 was considered as statistically significant. Kaplan–Meier analysis was also used for RFS analysis. For Univariate analysis of significance, the long rank test or Multivariate Cox analysis MedCalc Statistical Software version 16.4.3 (MedCalc Software bv, Ostend, Belgium; https://www.medcalc.org, accessed on 16 October 2021) was used. *p* < 0.05 was considered as statistically significant.

### 2.8. Availability of Data

Data is available upon request.

## 3. Results

### 3.1. Detection of CTCs in the Blood of NMIBC Patients

All 50 patients enrolled in this study had histologically confirmed diagnosis of NMIBC. The list of patients’ characteristics, including average age, sex, grade, T stage classification, histology, infiltration, recurrence and therapy, is shown in Table 1.

CTCs were isolated from blood samples in patients undergoing TURBT. Among the 50 NMIBC patients, 2 patients were not considered since they underwent BCG or BCG plus Mitomycin C instillations. Then, we found that among 48 NMIBC patients collected by ScreenCell filtration, 5 samples were CTCs negative, whereas 43/48 (89.6%) had detectable CTCs, with a median CTC count of about 4.0 (range 2–52). The CTC phenotype was directly evaluated by ddPCR, by analyzing the EpCAM and CK expression in all 43 filtered samples (Table 2). Data, normalized to β-actin concentration, were evaluated using the QuantaSoft Software and expressed as ratio between cDNA copies/μL of the variable and β-actin. Digital droplet PCR is highly sensitive (at level of single cells), highly specific, does not require personal interpretation, permits the identification of both EpCAM positive or negative CTCs and also simultaneously allows us to check the CTC purity, censoring the samples exceeding the cut-off for blood contaminant cells.

Data, normalized to *β-actin* concentration, were evaluated using the QuantaSoft Software and expressed as ratio between cDNA concentration of gene target and *β-actin*. Here we found that 40/43 (about 93%) of the CTCs examined were EpCAM-positive and CK-positive; 3 of 43 (7.0%) CTCs were EpCAM-positive but CK-negative and consequently were censored as non-conventional CTC (Table 3).

**Figure 1:**
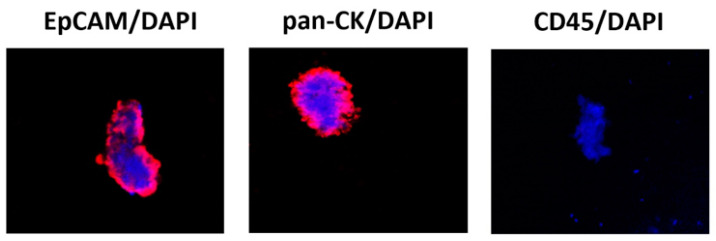
Confocal microscopy representative images of isolated CTCs stained for epithelial and hematopoietic markers. Representative image by confocal microscopy of circulating cancer cells stained with anti-human EPCAM, anti-human pan-CK or anti-human CD45 Abs followed by goat anti-mouse secondary antibody Alexa 594-conjugated. DAPI was used to counteract nuclei. Magnification 100×. CK = cytokeratin, an epithelial cytoplasmic marker; DAPI = nuclear marker; EpCAM = epithelial membrane marker; CD45 = leukocyte common antigen.

The CTC phenotype was further evaluated by confocal microscopy for the expression of EpCAM, pan-CK, CD45 and DAPI (Figure 1).

Recurrence is the major problem of NMIBC. EMT has been suggested to identify patients at high risk of developing a progressive disease in pTa [15] and the role of PD-L1 in BC recurrence has been suggested [2].

**Figure 2:**
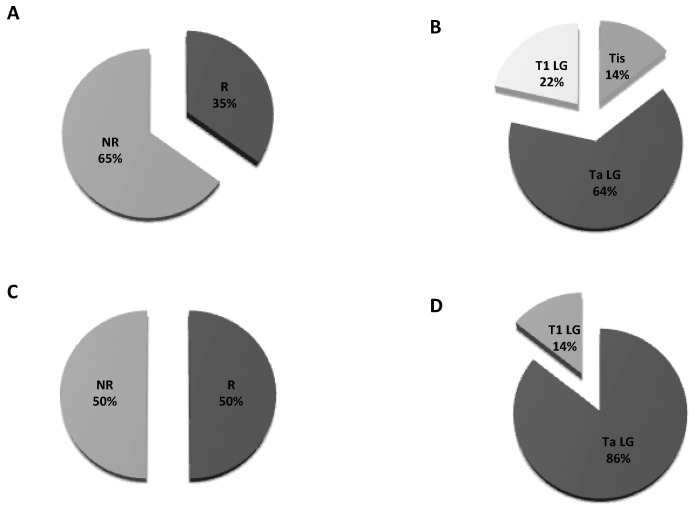
Pie charts of proportions for recurrence: (**A**) Patients’ distribution at the first follow up; (**B**) T stage distribution in patients developing recurrence at the first follow up; (**C**) patients’ distribution at the second follow up; (**D**) T stage distribution in patients developing recurrence at the second follow up. R = recurrent; NR = non recurrent.

Herein, we found 14 cases of recurrence out of all 40 CTC-positive patients at the first follow up: two were Tis, nine were Ta and three were T1 (Figure 2A,B). At the second follow up, among 14 patients, 7 out of 10 experienced recurrence (Figure 2C). The proportion of Ta to Tis was six out of seven vs. one out of seven, respectively (Figure 2D). Six out of seven patients who were positive at the second follow-up were already positive at the first.

### 3.2. Detection of Different EMT Markers in CTCs from NMIBC Patients

The expression of the EMT markers EpCAM and Vimentin (VIM) was evaluated in NMIBC patients by ddPCR and ROC analysis. We found that 35% and 45% of NMIBC samples showed low EpCAM or VIM expression, respectively (Table 4).

Based on the EMT markers, we also demonstrated that 17.5% of NMIBC patients displayed epithelial CTCs (E-CTCs), 55% hybrid (H-CTCs) EPCAM^High^/VIM^High^ or EPCAM^Low^/VIM^Low^ and 27.5% displayed mesenchymal CTCs (M-CTCs) (Table 5). We then analyzed the distribution of patients, according to the T pathological stage (Tis, Ta and T1). We found that of 31 pTa patients, about 16% and 29% were E-CTCs or M-CTCs and 55% were H-CTCs; of six pT1 patients, 33% were E-CTCs and 67% were H-CTCs; of three Tis patients, 33% were H-CTCs and 66% wee M-CTC (Table 5).

### 3.3. Gene Expression Profile of the PD-L1 and EMT Genes in Blood CTCs from NMIBC Patients

**Figure 3:**
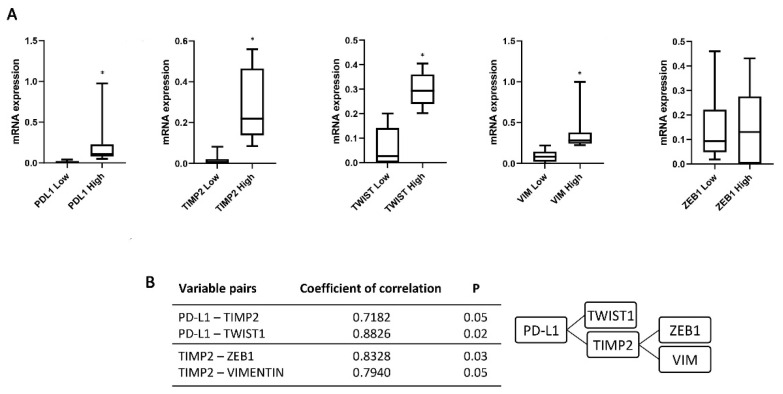
Expression of PD-L1, TIMP2, TWIST1, ZEB1 and VIM in CTCs isolated from NMIBC patients. (**A**) The expression was evaluated by ddPCR in NMIBC patients stratified according the recurrence at the first follow up. Expression values are presented as median with interquartile ranges (boxplot). *p* value was based on the Mann-Whitney U test, * *p* < 0.0001. (**B**) Correlation index evaluated for *PD-L1-TIMP2*, *PD-L1-TWIST1*, *TIMP2-ZEB1* and *TIMP2-VIM* variable pairs.

To evaluate the relation between the recurrence and the expression of PD-L1 and the EMT genes, the zinc finger E-box binding homebox 1 (ZEB1), Twist family BHLH transcription factor 1 (TWIST1), VIM and the invasive gene TIMP Metallopeptidase Inhibitor 2 (TIMP2) was evaluated. We stratified each marker expression patients in Low and High based on ROC analysis. PD-L1, TWIST1, VIM and TIMP2 expression levels showed a statistically significant difference in High respect Low-expression patients (Figure 3A). Moreover, a correlation index of studied genes confirmed the presence of a strong association between two different but related cluster genes: PD-L1 and TWIST1/TIMP2 and TIMP2 and ZEB1/VIM (*p* = 0.05 and 0.02 and *p* = 0.03 and 0.05, respectively) (Figure 3B). Thus, high PD-L1, TWIST1, VIM, ZEB1 and TIMP2 expression represents a specific gene signature in CTCs from NMIBC patients.

**Figure 4:**
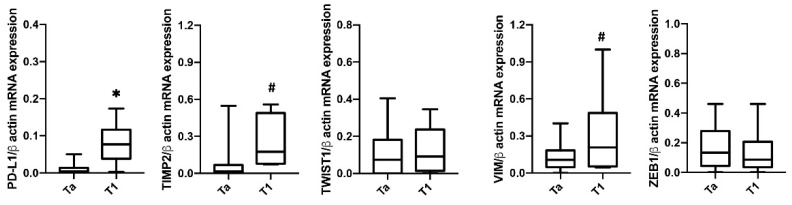
Expression of *PD-L1*, *TIMP2*, *TWIST1*, *ZEB1* and *VIM* in CTCs isolated from NMIBC patients according the T stage. The expression was evaluated by ddPCR. Expression values are presented as median with interquartile ranges (boxplot). *p* value was based on the Mann-Whitney U test for *PD-L1*, *TWIST1*, *ZEB1* and *VIM. TIMP2* was analyzed by Welch’s test. * *p* < 0.001, ^#^ *p* < 0.05.

Furthermore, we analyzed the expression of PD-L1 mRNA in CTCs based on pathological pTa and pT1 stage (Figure 4). Higher PD-L1 expression was found in pT1 (22.5%, *n* = 6/37) compared to pTa (77.5%, *n* = 31/37) NMIBC. Higher TIMP2 and VIM mRNA levels were found in pathological pT1 compared to pTa tumors in blood CTCs from NMIBC patients. No significant correlation was found for ZEB1 and TWIST1 markers.

### 3.4. Correlation between PD-L1, EMT Genes and Recurrence-Free Survival (RFS) in CTCs from NMIBC Patients

Firstly, we scrutinized the correlation between clinicopathological features and RFS time to determine their prognostic significance by Kaplan–Meier (Table 6) and Univariate analyses (Table 7). No statistical significance was found among age, grade, T stage, sex and histology.

**Figure 5:**
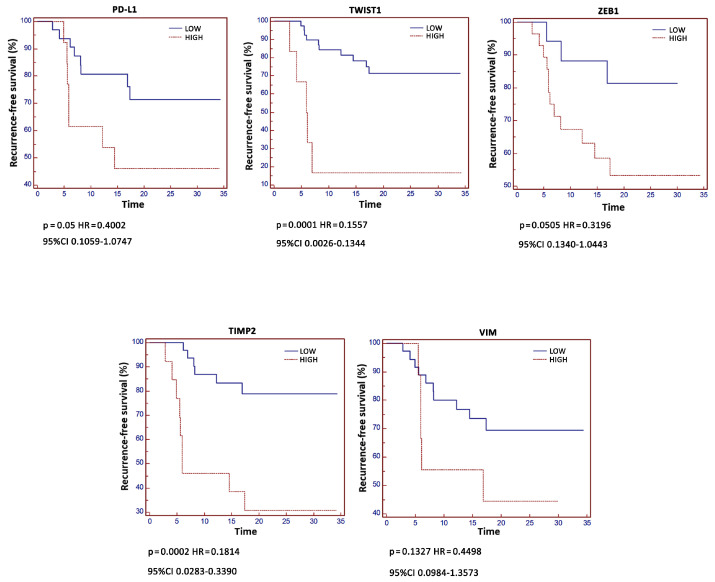
Kaplan–Meier analysis according to gene expression. Kaplan–Meier analysis was evaluated stratifying patients in High and Low expressing according to *PD-L1*, *TIMP2*, *TWIST1*, *VIM* and *ZEB1* levels.

In addition, the relation between RFS and *PD-L1*, *VIM*, *TWIST1*, *ZEB1* and *TIMP2* levels was carried out by Kaplan–Meier analysis (Figure 5). A significant correlation between high *PD-L1*, *TWIST1*, *ZEB1* and *TIMP2* and reduced RFS was evidenced.

Univariate analysis confirmed that high *PD-L1*, *TWIST1* and *TIMP2* were significantly associated with shorter tumor recurrence (*p*-value < 0.03, < 0.02 and < 0.05, respectively). Moreover, by Multivariate analysis using Cox’s proportional hazard model, high *PD-L1* (*p* < 0.01), *TWIST1* (*p* < 0.03), *TIMP2* (*p* = 0.11) expression were independent and significant prognostic factors for tumor recurrence in NMIBC patients (Table 8).

## 4. Discussion

Circulating tumor cells are usually assumed to be surrogates for micrometastatic disease. In BC, the risk of metastatic disease is clearly correlated to tumor stage. Indeed, the prognostically worse value of CTC has been demonstrated in MIBC and advanced urothelial BC [24,25]. CTCs represent an important circulating biomarker in NMIBC and were associated with disease recurrence and progression [26,27], although the mechanisms linking CTCs in the peripheral blood and local tumor recurrence in the bladder are still unknown.

In this study, we demonstrated that 90% of NMIBC patients showed detectable CTCs, with a median CTC count of about four. By ScreenCell filtration method and ddPCR, as well as confocal microscopy analysis, the 93% showed a canonical *EpCAM+ /CK+ CD45−* CTC phenotype. Higher *PD-L1*, *TWIST1*, *VIM*, *ZEB1* and *TIMP2* expression was evidenced in pT1 NMIBC patients, compared to pTa samples. Moreover, a strong association between *PD-L1* and *TWIST1* and *TIMP2* and between *TIMP2* and *ZEB1* and *VIM* was evidenced by correlation index analysis.

Stratification of NMIBC patients into low and high *PD-L1*, *TWIST1*, *VIM*, *ZEB1* and *TIMP2* expression by ROC analysis permits the estimation of the risk of recurrence and progression, and it is also essential for decisions about adjuvant intravesical instillation [2]. Herein, by Kaplan–Meier analysis, we found that RFS was reduced in patients with high *PD-L1* (*p* = 0.05, HR = 0.4002, 95% CI = 0.1059–1.0747) as well as *TWIST1* (*p* = 0.0001, HR = 0.1557, 95% CI = 0.0026–0.1344), *ZEB1* (*p* = 0.0505, HR = 0.3196, 95% CI = 0.1340–1.0443) and *TIMP2* (*p* = 0.0002, HR = 0.1814, 95% CI = 0.0283–0.3390) mRNA expression compared to those expressing low levels (6.5 vs. 17.4, 6.0 vs. 17.4, 6.9 vs. 17.4, 5.5 vs. 17.4 and 5.9 vs. 16.9 months, respectively). Similar results were obtained for *PD-L1*, *TWIST1* and *TIMP2* (*p* = 0.03, 0.02 and 0.05, respectively), by univariate analysis. Finally, by multivariate analysis, high *PD-L1* and *TWIST1* expression was found to be independent and negative prognostic factors for tumor recurrence in NMIBC patients.

The prognostic significance of *PD-L1* in BC has been associated with recurrence and poor survival [2]. However, data on *PD-L1* expression and its prognostic significance are controversial. Positive and high *PD-L1* levels were found in 46% and 7% of NMIBC-HG, with the positive expression associated with submucosal invasion and refractory-tumor recurrence. Moreover, after BCG treatment, 55% and 11% of NMIBC-HG patients had positive and high *PD-L1* levels [28]. Surprisingly, in T1-HG NMIBC patients, high *PD-L1* mRNA expression represented a favorable prognostic factor for better RFS in the absence of anti-PD-1 or anti-PD-L1 treatments [29]. Thus, this high *PD-L1* expression could be promising in the view of anti-PD-L1 therapies.

Similarly to our results, in ESCC, high *PD-L1* tumor levels are associated with EMT and poor prognosis. High *PD-L1* was associated with worse overall and relapse-free survival. Moreover, a correlation between *PD-L1*, *ZEB1* expression and poor prognosis was found, suggesting that a cooperative mechanism bridging tumor immune avoidance and EMT contributes to tumor malignancy in ESCC [11].

In cancer cells, EMT-induced signals may be the results of EMT-transcription factors, including *ZEB1* and *TWIST1* [30]. The *PD-L1* gene promoter region contains a *ZEB1* binding site, which has led to speculation that *PD-L1* expression may be regulated by the *ZEB1* transcription factor. *TWIST1* and *VIM* correlate with recurrence in NMIBC patients [31,32]. Higher *TIMP2* mRNA expression in superficial BC patients with recurrence, compared with those without recurrence, was found. In addition, Cox’s multivariate analysis revealed that elevated *TIMP2* was associated with a high incidence of intravesical recurrence [33]. Since CTCs are implicated in the metastatic spread, and in NMIBC they are associated with recurrence, it is intriguing that there is a high expression of invasive marker *TIMP2* in recurrent NMIBC patients.

Overall, the high *PD-L1* and EMT/invasive expression in blood CTCs from NMIBCs could identify patients with a super-high-risk of recurrence and progression. Moreover, it may change the treatment of super-high-risk NMIBC patients who have already a systemic disease at diagnosis and might therefore potentially benefit from systemic treatment with immune checkpoint inhibitors.

## 5. Conclusions

Our findings demonstrate the ability to detect CTCs in blood from NMIBC patients’ and quantify the *PD-L1*, *TWIST1*, *VIM*, *ZEB1* and *TIMP2* mRNA expression using the ddPCR assay, showing high specificity and sensitivity. Exploration analysis of RFS suggests a trend toward worse recurrence time in those patients with high *PD-L1*, *TWIST1*, *ZEB1* and *TIMP2* mRNA expression, strongly supporting the necessity to consider NMIBC patients as ideal candidates for systemic approaches with immune checkpoint inhibitors. While the data presented here are compelling, it should be underlined that the principal limitation of this study was the small sample size and requires validation with a larger study encompassing a broader patient population. Nonetheless, these data provide initial support for the broader development of *PD-L1* and EMT CTC signature expression. With further study, the *PD-L1/TWIST1/TIMP2* expression in CTCs isolated from peripheral blood could become new prognostic and predictive biomarkers to stratify treatment and to predict immunotherapy responses in low-grade NMIBCs.

## Figures and Tables

**Table 1:** Patients’ demographic and clinical characteristic and classification based on WHO 2017 criteria.

Data	Patients
**Gender, *n* (%)**	
Male	41 (82%)
Female	9 (18%)
**Age years**	
Range	(47–93)
Media	73.2
Median	74
**Tumor histology** Papillary	37 (74%)
Not Papillary	13 (26%)
**Pathological Tumor Grade**	
Low	40 (80%)
High	10 (20%)
**Pathological Tumor Stage**	
Tx	1 (2%)
Tis	3 (6%)
Ta	36 (72%)
T1	10 (20%)
**Recurrence**	
Yes	20 (40%)
No	30 (60%)
**Tissue Infiltration**	
Yes	5 (10%)
No	45 (90%)
**Instillations**	
Yes (BCG or BCG plus MitC)	2 (4%)
No	48 (96%)

**Table 2:** Gene expression evaluated by ddPCR. Bold: ID patients.

	EPCAM	CK		EPCAM	CK
**NMIBC 1**	0.2458	0.31145	**NMIBC 23**	0.10238	0.06905
**NMIBC 2**	0.1179	0.16667	**NMIBC 24**	0.34483	0.16034
**NMIBC 3**	0.4273	0.33752	**NMIBC 25**	0.0416	0.00000
**NMIBC 4**	0.0315	0.36292	**NMIBC 26**	0.0691	0.05647
**NMIBC 5**	0.2771	0.11547	**NMIBC 27**	0.1963	0.12313
**NMIBC 6**	0.344	0.13298	**NMIBC 28**	0.2425	0.14434
**NMIBC 7**	0.1339	0.0324	**NMIBC 29**	0.0484	0.04449
**NMIBC 8**	0.4008	0.2776	**NMIBC 30**	0.0516	0.01572
**NMIBC 9**	0.1803	0.03673	**NMIBC 31**	0.07946	0.04948
**NMIBC 10**	0.0858	0.11489	**NMIBC 32**	0.02271	0.15895
**NMIBC 11**	0.2591	0.22878	**NMIBC 33**	0.41441	0.20751
**NMIBC 12**	0.50918	0.28367	**NMIBC 34**	0.095	0.12333
**NMIBC 13**	0.05625	0.04	**NMIBC 35**	0.22199	0.12474
**NMIBC 14**	0.6005	0.04381	**NMIBC 36**	0.01373	0.00000
**NMIBC 15**	0.09119	0.20061	**NMIBC 37**	0.17112	0.24293
**NMIBC 16**	0.02533	0.06267	**NMIBC 38**	0.21712	0.08065
**NMIBC 17**	1.27187	0.11111	**NMIBC 39**	0.07692	0.15858
**NMIBC 18**	0.1788	0.49721	**NMIBC 40**	0.17618	0.10277
**NMIBC 19**	0.1521	0.07606	**NMIBC 41**	0.20774	0.12194
**NMIBC 20**	0.3	0.18844	**NMIBC 42**	0.37625	0.27421
**NMIBC 21**	0.02426	0.05391	**NMIBC 43**	0.12639	0.00000
**NMIBC 22**	0.052	0.07067			

**Table 3:** Blood patients’ CTCs.

Percentage CTCs Positive after Cell Screen Filtration	*n* (%)
Positive	43 (89.6%)
Negative	5 (10.4%)
**CTC specificity after ddPCR**	
EpCAM + /CK + /CD45-	40 (93.0%)
EpCAM + /CK − /CD45-	3 (7.0%)
EpCAM − /CK + /CD45-	0 (0.0%)
**Patients pathological grade and stage**	***n* (%)**
Total	40 (100%)
Tis	3 (7.5%)
Ta LG	27 (67.5%)
Ta HG	4 (10.0%)
T1 LG	6 (15.0%)
T1 HG	0 (0.0%)

**Table 4:** Correlation between EpCAM or VIM expression levels in blood CTCs and pathological stage T in NMIBC patients.

EpCAM^low^	(*n* = 14/40, 35.0%)
Tis	1/14 (7.1%)
Ta	9/14 (64.3%)
T1	4/14 (28.6%)
**EpCAM^high^**	**(*n* = 26/40, 65.0%)**
Tis	2/26 (7.7%)
Ta	22/26 (84.6%)
T1	2/26 (7.7%)
**VIM^low^**	**(*n* = 18/40, 45.0%)**
Tis	3/18 (16.7%)
Ta	13/18 (72.2%)
T1	2/18 (11.1%)
**VIM^high^**	**(*n* = 22/40, 55.0%)**
Ta	18/22 (81.8%)
T1	4/22 (18.2%)

**Table 5:** NMIBC patient distribution (*n* = 40) according to the different EMT phenotype and pathological T stage in blood CTCs.

T	E-CTCs	H-CTCs	M-CTCs
Tis (*n* = 3, 7.5%)	0 (0.0%)	1 (33.3%)	2 (66.7%)
Ta (*n* = 31, 77.5%)	5 (16.1%)	17 (54.8%)	9 (29.1%)
T1 (*n* = 6, 15.0%)	2 (33.3%)	4 (66.7%)	0 (0.0%)

E-CTCs, Epithelial-CTC = EpCAM^low^/VIM^high^, H-CTC, Hybrid CTCs = EpCAM^low^/VIM^low^; EpCAM^high^/VIM^high^; M-CTC, Mesenchymal CTC = EpCAM^high^/VIM^low^.

**Table 6:** Correlation analysis by Kaplan-Meier between patients’ characteristics and RFS.

Variables	*n*	*p*-Value	HR	95% CI	Chi-Square
Age: <60 vs. >60 years >60 years = 29/40 (72.5%) vs. <60 years = 11/40 (27.5%)	40	=0.2355	0.4168	0.1545–1.5827	-
Pathological Grade: Low vs. High Low Grade = 36/40 (90%) vs. High Grade = 4/40 (10%)	40	=0.1719	0.3344	0.1143–1.1234	-
T stage: Tis vs. Ta vs. T1 Tis = 3/40 (7.5%) vs. Ta = 31/40 (77.5%) vs. T1 = 6/40 (15.0%)	40	=0.1019		-	4.5680
Sex: Female vs. Male Female = 7/40 (17.5%) vs. Male = 33/40 (82.5%)	40	=0.6378	0.6987	0.1879–2.7854	-
Histology: Papillary vs. Not Papillary Papillary = 29/40 (72.5%) vs. Not Papillary = 11/40 (27.5%)	40	=0.0832	2.4631	0.8657–10.3978	-

**Table 7:** Univariate analysisof clinicopathological characteristics affecting RFS in NMIB patients.

Variables	*n*	*p*-Value	Hazard Ratio (95% CI)
Age: <60 vs. >60 years	40	=0.2594	0.5335–10.5083
Pathological Grade: Low vs. High	40	=0.8370	0.2634–5.2006
T stage: Tis vs. Ta vs. T1	40	= 0.1679	0.8390–2.7830
Sex: Female vs. Male	40	=0.6511	0.3159–6.3487
Histology: Papillary vs. Not Papillary	40	=0.0900	0.1389–1.1474

**Table 8:** Univariate and multivariate Cox proportional hazards regression analysis for recurrent free survival (RFS) in NMIB patients.

Molecular Variables	Univariate Analysis		Multivariate Analysis	
	Hazard Ratio (95% CI)	*p*-Value	Hazard Ratio (95% CI)	*p*-Value
PD-L1	1.0721–6.8003	<0.03 *	1.3267–9.5962	<0.01 *
TWIST1	0.5286–3238.7085	<0.05 *	1.4868–8518.2648	<0.03 *
TIMP2	1.5390–133.7713	<0.02 *	0.6190–128.9769	=0.11
VIM	0.2926–27.0342	<0.33	*ns*	*ns*
ZEB1	0.1792–159.9790	<0.36	*ns*	*ns*

*ns* = non statistically significant; * *p* < 0.05.

## Data Availability

The data that support the findings of this study are available from the corresponding authors upon request.
